# Sorafenib continuation or discontinuation in patients with unresectable hepatocellular carcinoma after a complete response

**DOI:** 10.18632/oncotarget.4076

**Published:** 2015-06-03

**Authors:** Yingqiang Zhang, Wenzhe Fan, Kangshun Zhu, Ligong Lu, Sirui Fu, Jinhua Huang, Yu Wang, Jianyong Yang, Yonghui Huang, Wang Yao, Jiaping Li

**Affiliations:** ^1^ Department of Interventional Oncology, The First Affiliated Hospital, Sun Yat-sen University, Guangzhou, China; ^2^ Department of Interventional Radiology, The Third Affiliated Hospital, Sun Yat-sen University, Guangzhou, China; ^3^ Department of Interventional Oncology, Guangdong General Hospital, Guangzhou, China; ^4^ Department of Medical Imaging and Interventional Radiology, Sun Yat-sen University Cancer Center & State Key Laboratory of Oncology in Southern China, Guangzhou, China; ^5^ Department of Interventional Radiology, The First Affiliated Hospital, Sun Yat-sen University, Guangzhou, China

**Keywords:** hepatocellular carcinoma, transarterial chemoembolization, radiofrequency ablation, complete response, sorafenib

## Abstract

**Aims:**

To assess the efficacy of continued administration of sorafenib for patients with unresectable hepatocellular carcinoma (HCC) treated with local regional therapy (LRT) after a complete response (CR), also, the adverse events of sorafenib after discontinuation of administration were observed.

**Methods:**

Between April 2008 and May 2012, 956 consecutive patients with unresectable HCC treated with LRT (transarterial chemoembolization, radiofrequency ablation) combined with sorafenib were retrospectively investigated. Of these, 157 patients with a CR were enrolled: 102 of them continued to receive sorafenib (test group) and the other 55 stopped receiving sorafenib (control group).

**Results:**

The median recurrence-free survival (RFS), post-complete response overall survival (pOS) and overall survival (OS) in the test and control groups were 11 months (95% CI: 6.1, 15.9), 25 months (95% CI: 20.7, 29.3) and 33 months (95% CI: 29.2, 36.8) and 12 months (95% CI: 10.4, 13.6), 28 months (95% CI 24.2, 31.8) and 34 months (95% CI: 30.8, 37.2) respectively. The differences in RFS, pOS and OS between the groups were not significant (*P* = 0.768, 0.797 and 0.730, respectively). The adverse events related to sorafenib resolved after discontinuation of administration and the quality of life (QoL) scores improved.

**Conclusions:**

Patients with unresectable HCC who achieved a CR did not benefit from continued sorafenib in terms of RFS, pOS or OS. The adverse events of sorafenib were reversible, and discontinuation of sorafenib may improve the QoL of patients who have achieved a CR.

## INTRODUCTION

Hepatocellular carcinoma (HCC) is the fifth most common malignancy and the third leading cause of cancer-related death worldwide, and its incidence is increasing [1]. Liver resection, liver transplantation, and percutaneous ablation are the main radical treatments for HCC. Unfortunately, only 30–40% of early stage patients (*Barcelona Clinic Liver Cancer* BCLC stage A) are amenable for such curative therapies, and more than 50% of all HCCs are diagnosed at an unresectable tumor stage with a median overall survival of 11–20 months [2, 3].

Transarterial chemoembolization (TACE) and radiofrequency ablation (RFA) are the most common local regional treatments (LRT) for most patients with unresectable HCC [2–5]. Moreover, the combination of TACE and RFA is more effective than TACE or RFA alone for the treatment of patients with large HCC exceeding 3.5 cm [6, 7]. However, after TACE, the residual cancer cells are in an extensive hypoxic or even anoxic environment. Hypoxia can lead to adaptive responses with VEGF overexpression [8], which may lead to tumor growth, invasion and metastasis. Sorafenib, an oral inhibitor of multiple kinases involved in HCC proliferation and angiogenesis, is recommended for advanced HCC [2, 3, 9, 10].

Recently, there has been increasing focus on LRT combined with sorafenib to potentially improve the efficacy for patients with unresectable HCC [11–14]. However, these clinical studies did not address whether sorafenib is effective in the few patients who have achieved a complete response (CR). In our clinical practice, a few patients with unresectable HCC who treated with LRT have achieved a CR are continued on sorafenib. This is made possible by a program supported by the China Charity Federation that funds continued sorafenib treatment after the first 3 months for patients who benefit from its initial administration. Without the additional financial burden, most patients prefer to continue sorafenib administration even if they have achieved a CR. However, when a tumor has achieved a CR, regular surveillance is usually performed rather than continued treatment in clinical practice.

However, the STORM study [15] (Sorafenib as Adjuvant Treatment in the Prevention of Recurrence of Hepatocellular Carcinoma) found that the administration of sorafenib to HCC patients after potentially curative treatments conferred no benefits in terms of recurrence-free survival (RFS) or time to recurrence, the study cohort consisted of BCLC stage A patients who had undergone curative therapy. It is well known, recurrence rate of unresectable stage HCC treated with LRT is significantly higher than that of early stage HCC after curative treatment [2, 3]. Therefore, it is important to clarify the efficacy of continued sorafenib in patients who had unresectable stage HCC and high recurrence risk.

We conducted a retrospective study of 157 patients with unresectable HCC who had achieved a CR and compared the RFS, post-complete response overall survival (pOS) and overall survival (OS) between patients with continued sorafenib treatment and patients without sorafenib treatment after a CR. The adverse events of sorafenib after discontinuation were also observed.

## RESULTS

### Study population

**Figure 1:**
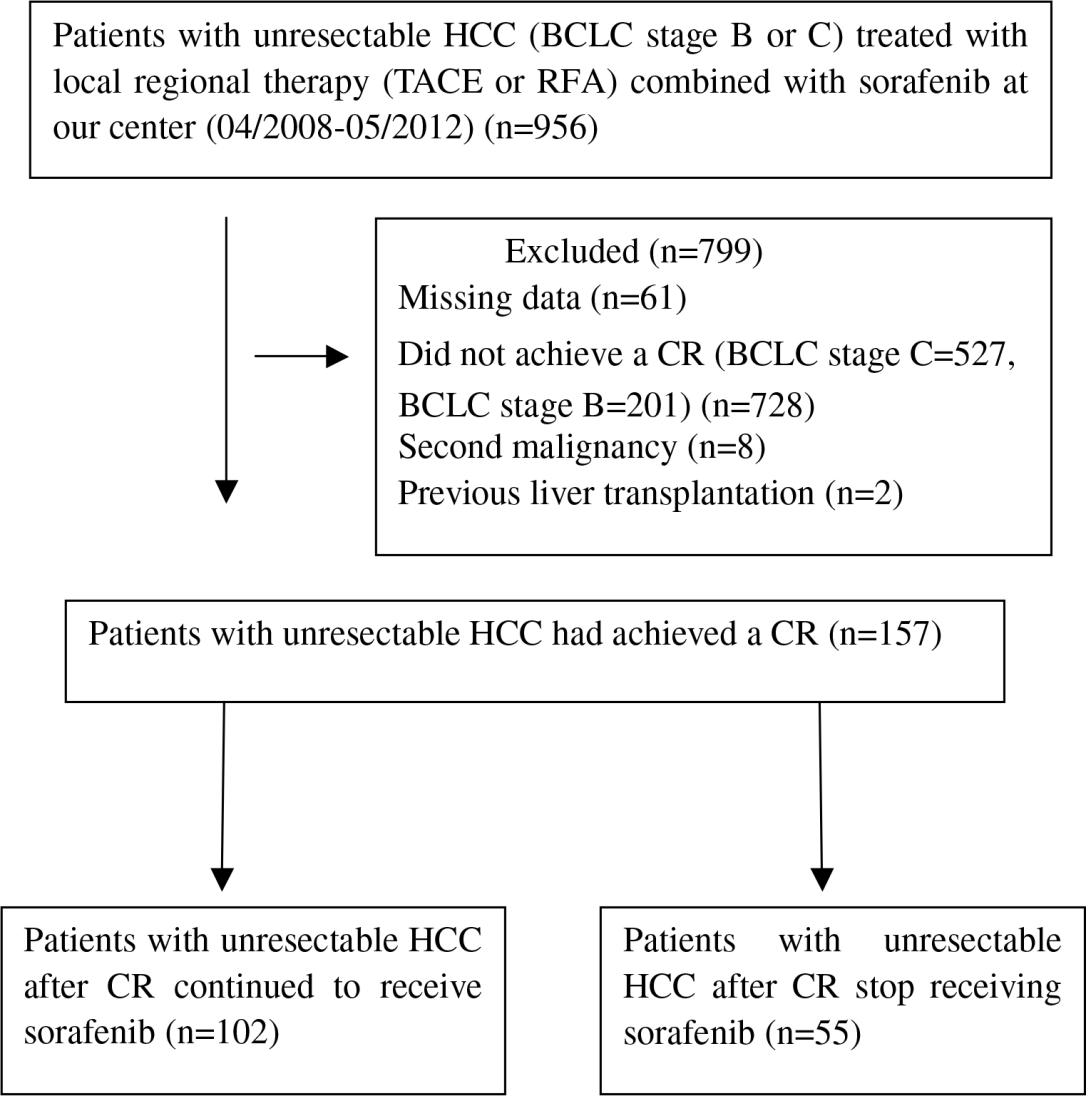
Flow diagram shows patient selection HCC, hepatocellular carcinoma; BCLC, Barcelona Clinic Liver Cancer; TACE, transarterial chemoembolization; RFA, radiofrequency ablation; CR, complete response.

In total, between April 2008 and May 2012, 956 consecutive patients with unresectable HCC treated with LRT (TACE, RFA) combined with sorafenib were retrospectively observed during the study period. A total of 799 patients were excluded from the study because they met the exclusion criteria. As a result, 157 patients who had achieved a CR were enrolled in this study, 102 of them continued to receive sorafenib (test group) and the other 55 patients the administration of sorafenib was stopped (control group) (Figure 1). The baseline characteristics of all patients are shown in Table 1. These did not differ significantly between the two groups in terms of the cause of liver disease, liver function, tumor characteristics or previous therapy. The majority of the patients were male and hepatitis B virus infection was the most common underlying disease. All of patients were at BCLC stage B.

**Table 1:** Comparison of baseline patient characteristics

Characteristics	Test group (*n* = 102)	Control group (*n* = 55)	*P*
Age (years)	52.7 ± 12.2	56.4 ± 9.5	0.051
Sex			1.000
Male	99(97.1)	53(96.4)	
Female	3(2.9)	2(3.6)	
HBsAg			1.000
Present	97(95.1)	52(94.5)	
Absent	5(4.9)	3(5.5)	
Cirrhosis			0.945
Yes	83(81.4)	45(81.8)	
No	19(18.6)	10(18.2)	
Prior surgical			0.857
Yes	21(20.6)	12(21.8)	
No	81(79.4)	43(78.2)	
No. of tumors			0.306
1–3	29(28.4)	20(36.4)	
>3	73(71.6)	35(63.6)	
Size of main tumor (cm)	7.3 ± 3.9	6.3 ± 3.9	0.131
Size range of tumor (cm)			0.591
≥5	62(60.8)	31(56.4)	
<5	40(39.2)	24(43.6)	
Child-Pugh class			1.000
A	99(97.1)	53(96.4)	
B	3(2.9)	2(3.6)	
BCLC stage			-
B	102	55	
C	0	0	
AFP (ng/mL)			0.221
<20	16(15.7)	13(23.6)	
>20	86(84.3)	42(76.4)	

Note- data are numbers of patients, data in parentheses are percentages. HBsAg, hepatitis B surface antigen; BCLC, Barcelona Clinic Liver Cancer; AFP, alpha-fetoprotein.

### Treatment

The median duration of sorafenib administration before CR in the test and control groups was 5.4 months (range, 1–9) and 5.0 months (range, 0.5–9), respectively, The median duration of sorafenib treatment after CR in the test group was 25.4 months (range, 12–55). The median number of TACE procedures per patient in the test and control groups were 6.2 (range, 1–12) and 4.7 (range, 2–10), respectively, and the median number of RFA procedures per patient in the test and control groups was 0.6 (range, 0–3) and 0.8 (range, 0–4), respectively.

### Survival

At the end of follow-up (December 2013), 72 (70.6%) patients in the test group and 40 (72.7%) patients in the control group had died. The median follow-up for these patients was 34.6 ± 11.8 months (range, 22–68) and the total follow-up time after initial therapy was more than 5 years. The causes of death are listed in Table 2.

**Table 2:** Causes of death between the test and control groups

Causes of death	Test (*n* = 102)	Control (*n* = 55)	*P*
Tumor progression	48	21	0.285
Liver failure with stable tumor	20	15	0.271
Other	4	4	0.596

**Figure 2:**
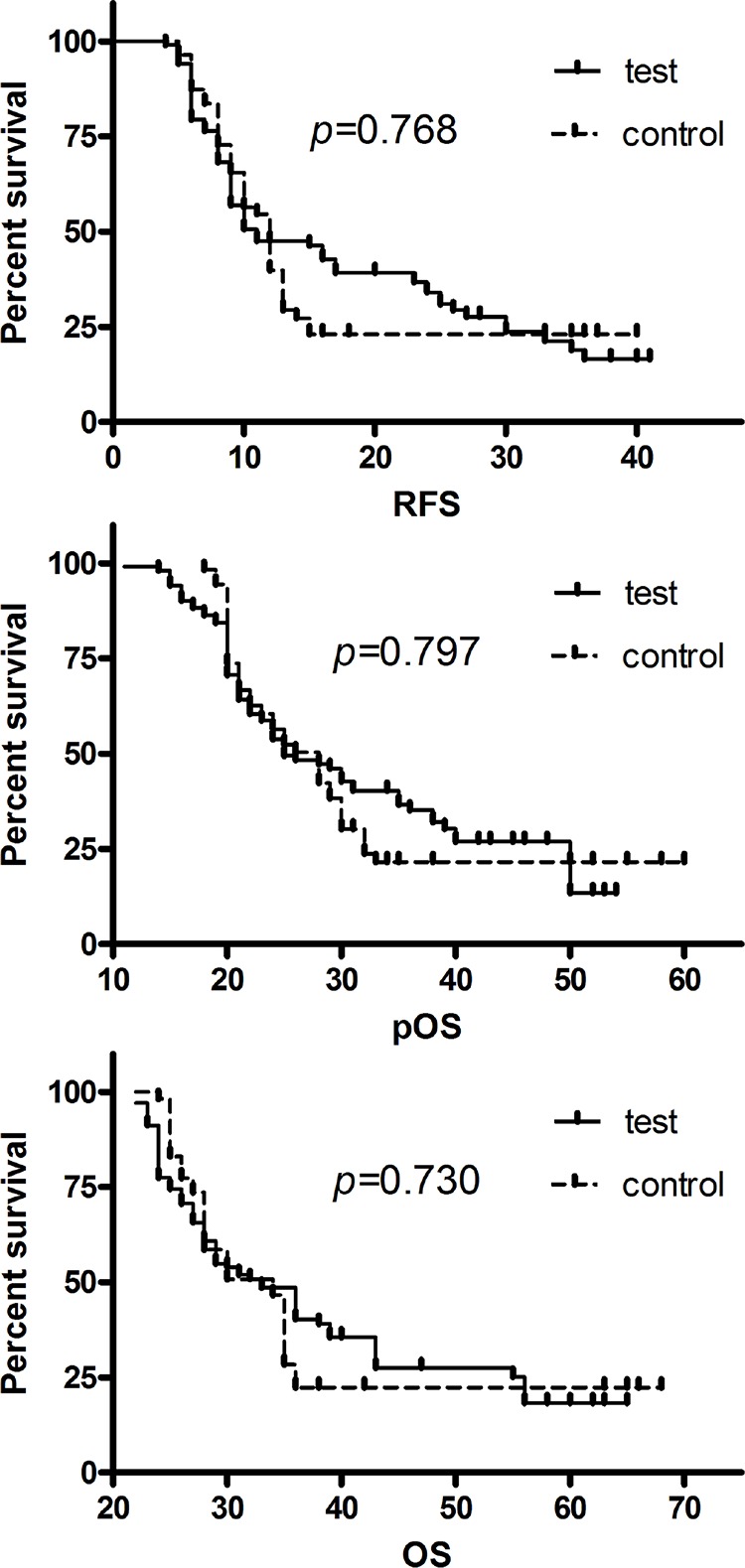
**A.** Recurrence-free survival (RFS) curves of patients with unresectable HCC for the test and control groups. Median RFS for test group = 11 months; median RFS for control group = 12 months; *P* = 0.768. **B.** Post-complete response overall survival (pOS) of patients with unresectable HCC for the test and control groups. Median pOS for test group = 25 months; median pOS for control group = 28 months; *P* = 0.797. **C.** Overall survival (OS) of patients with unresectable HCC for the test and control groups. Median OS for test group = 33 months; median OS for control group = 34 months; *P* = 0.730.

Recurrence free survival, The median RFS was 11 months in the test group (95% CI: 6.1, 15.9) and 12 months in the control group (95% CI: 10.4, 13.6); this difference was not significant. (*P* = 0.768; Figure 2A).

Post-complete response overall survival, The median pOS was 25 months (95% CI 20.7, 29.3) in the test group and 28 months (95% CI 24.2, 31.8) in the control group; this difference was not significant. (*P* = 0.797; Figure 2B).

Overall survival, The median OS was 33 months (95% CI 29.2, 36.8) in the test group and 34 months (95% CI 30.8, 37.2) in the control group; this difference was not significant. (*P* = 0.730; Figure 2C).

### Safety and toxicity

The adverse events related to treatment with TACE, RFA and sorafenib were comparable to those reported in the literature [9–14], and there were no deaths related to any of these treatments. The most common complications after TACE or RFA were abdominal pain (68.2%), fever (35.0%), vomiting (45.9%), temporary elevation of transaminase (58.6%), ascites (5.1%), pleural effusion (2.5%) and liver abscess (0.6%) (Table 3).

**Table 3:** Complications after treatment

Complication	Test group (*n* = 102)	Control group (*n* = 55)	*P*
Abdominal pain			0.594
Grade 1–2	71(69.6)	36(65.5)	
Fever(>38.5°C)			0.797
Grade 1–2	35(34.3)	20(36.4)	
Vomiting			0.794
Grade 1–2	46(45.1)	26(47.3)	
Temporary elevation of transaminase			0.794
Grade 1–2	59(57.8)	33(60.0)	
Pleural effusion			1.000
Grade 1–2	3(2.9)	1(1.8)	
Ascites			1.000
Grade 1–2	5(4.9)	3(5.5)	
Liver abscess			0.753
Grade 3–4	0	1(1.8)	

Note- data are numbers of patients, data in parentheses are percentages.

Sixteen temporary reductions in sorafenib dose were made because of toxicity in the test group and 5 in the control group, respectively. Sorafenib was discontinued permanently because of tumor progression and liver dysfunction in 21 patients in the test group. Three patients stop administrating sorafenib because of the adverse events in the control group. The others received the full dose of sorafenib without interruption due to toxicity. The most common adverse events related to sorafenib were hand-foot skin reaction (HFSR), alopecia, diarrhea, weight loss, fatigue, and hypertension. The most common grade 3 or higher adverse events were HFSR, diarrhea and hypertension. Before the discontinuation, the incidence of side effects of sorafenib was similar between the two groups. However, two months after discontinuation, the most of side effects of sorafenib were resolved in the control group. The adverse events related to sorafenib are listed in Table 4.

**Table 4:** Adverse events related to sorafenib before and 2 months after discontinuation of sorafenib

	Before discontinuation			2 months after discontinuation	
Adverse events	Test (*n* = 102)	Control (*n* = 55)	*P*	Control (*n* = 55)	*P*
Hand-foot skin reaction	72(70.6)	36(64.5)	0.789	8(14.5)	<0.001
Grade 1–2	61(59.8)	31(56.4)		8(14.5)	
Grade 3–4	11(10.8)	5(9.1)		0	
Diarrhoea	52(50.9)	27(49.1)	0.853	0	<0.001
Grade 1–2	44(43.1)	24(43.6)		0	
Grade 3–4	8(7.8)	3(5.5)		0	
Hypertension	4(3.9)	2(3.6)	0.309	0	0.361
Grade 1–2	4(3.9)	1(1.8)		0	
Grade 3–4	0	1(1.8)		0	
Alopecia			0.345		0.004
Grade 1–2	39(38.2)	24(43.6)		10(18.2)	
Weight loss			0.924		0.039
Grade 1–2	40(39.2)	22(40)		12(21.8)	
Fatigue			0.797		0.002
Grade 1–2	35(34.3)	20(36.4)		6(10.9)	

Note- data are numbers of patients, data in parentheses are percentages.

**Figure 3:**
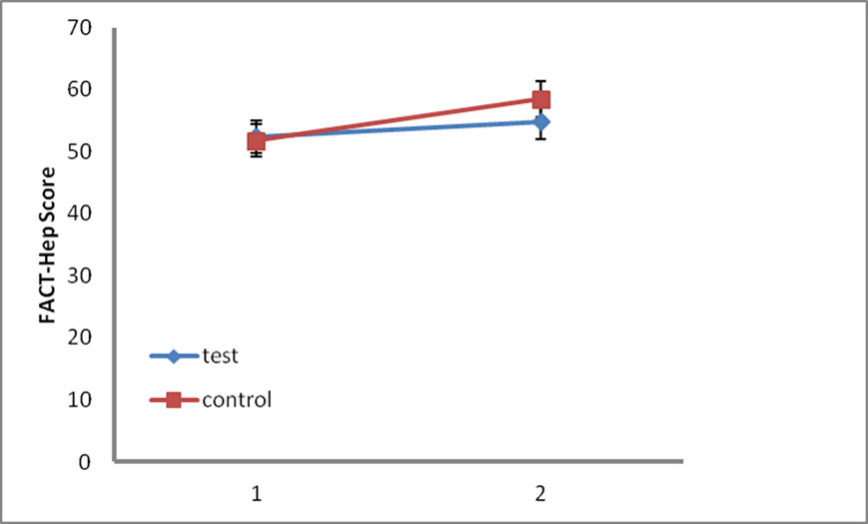
Quality-of-life assessments according to the 18-item hepatobiliary cancer subscale (HepCS) in functional assessment of cancer therapy-hepatobiliary (FACT-Hep) Dots and error bars indicate the mean total scores and 95% CI, respectively. Higher scores indicate a better quality of life. 1, one month after administration of sorafenib; 2, two months after the complete response.

QoL Symptom score questionnaire responses were collected from 154 (98.1%) of 157 patients at one month after administration of sorafenib, 146 patients (93.0%) at the 2 months after the discontinuation of sorafenib. There were no significant differences in QoL score between the two groups at one month after administration of sorafenib. However, at the 2 months after the CR, QoL scores were significantly higher in the control group (*P* < 0.001; Figure 3).

## DISCUSSION

We assessed whether sorafenib further improves the survival of patients with unresectable HCC who have achieved a CR. We also observed the adverse events related to sorafenib that occurred when administration was stopped. This is the first study to date to compare the efficacy of sorafenib for unresectable HCC in patients with CR. The data came from the center which had the largest population of liver cancer and considerable experience with TACE and RFA in the South China. This study also has the longest follow-up since sorafenib was introduced in 2008.

The modified Response Evaluation Criteria In Solid Tumors (mRECIST) [16] is the most used radiological evaluation for HCC. Sometimes evaluation of CR can be difficult and depends on the individual radiologist's experience, but also on the imaging equipment used, especially for larger tumors. Although serum alpha-fetoprotein (AFP) is not currently recommended as a diagnosis marker for HCC [2, 3], in patients with cirrhosis or chronic hepatitis B, AFP is an important indicator of diagnosis and prediction for HCC occurrence [17]. Therefore, we used radiological evaluation together with AFP evaluation to assess the efficacy of treatment; both radiological evaluation criteria and AFP normalization can be used to assess CR except in cases of HCC with negative serum AFP. Therefore, this may improve the power of evaluation of CR.

Interestingly, the results of this study showed that the median RFS, pOS and OS were slightly longer in controls than in the test group. However, this difference was not statistically significant. Therefore, we suggest that continued administration of sorafenib may not benefit patients with unresectable HCC who have had a CR. This contradicts studies showing the effectiveness of TACE with sorafenib for intermediate-advanced HCC [11–14], but might be explained by the inclusion of more advanced stage patients with fewer CRs in those studies. On the other hand, we confirm the results of another report [18] showing that TACE plus sorafenib achieved a comparable effectiveness to treatment with TACE alone for patients with HCC (most patients were BCLC stage A and B). Similarly, Cabrera et al. [12] reported that the combination of TACE and sorafenib achieved a median survival time of 18.5 months for patients with BCLC stage B. Clinical trial and meta-analysis data confirm that chemoembolization improves survival of BCLC stage B patients up to 19–20 months [3, 19]. Moreover, the phase III STORM study found that the administration of sorafenib to HCC patients after potentially curative treatments conferred no benefits in terms of RFS or time to recurrence [15].

There are two likely explanations for our findings. First, sorafenib blocks tumor cell proliferation by targeting Raf-MEK-ERK pathway and exerts tumor angiogenesis by targeting VEGFR-1, VEGFR-2, VEGFR-3, and PDGFR-β [9, 10], which is different from the molecular mechanism of hepatocarcinogenesis [20]. Second, acquired resistance to molecular target agent inevitably occurs in almost all tumors including non-small cell lung cancer (NSCLC) [21], and gastrointestinal stromal tumors (GIST) [22]. Therefore, a long duration of sorafenib for patients with unresectable HCC after CR may not be effective.

In addition, the quality of life is a priority in every disease management. Our results showed that the side effects of sorafenib, such as HFSR, diarrhea and hypertension, resolved after the discontinuation of sorafenib. Weight loss and alopecia also improved gradually. We suppose that these were the main causes of the improvement in the QoL score in the control group after the discontinuation of sorafenib.

This study has several limitations. First, it is a retrospective analysis and the sample size of control group is relatively small. Second, therapeutic options (continuation or discontinuation) in patients with unresectable HCC after a CR were individually determined by the attending physician, which likely led to selection bias in our population. However, the bias was limited by choosing similar baseline characteristics between the two groups. Third, the time point at which patients began to take sorafenib was not coincident, but this difference between the two groups was not significant. In this study, although patients with CR were downstaged, none of those were treated with surgical or liver transplantation. The main reasons were as follows: (i) most of the patients had multiple-nodules; (ii) a donor liver would have been insufficient for all HCC patients; and (iii) consideration of the complexity and cost of the liver transplantation procedure.

In conclusion, our findings suggest that there is no benefit in terms of RFS, pOS and OS in continuing sorafenib in patients with unresectable HCC who have achieved a CR. The side effects of sorafenib were reversible, and the quality of life of patients was improved after discontinuation of sorafenib. Further and larger prospective trials are needed to confirm this conclusion.

## MATERIALS AND METHODS

### Study design and patient selection

The study protocol was approved by the First Affiliated Hospital of Sun Yat-sen University ethics committees. Written informed consent was obtained from each participant in according with the Declaration of Helsinki. Between April 2008 and May 2012, consecutive patients with unresectable HCC treated with LRT (TACE, RFA) combined with sorafenib were retrospectively studied.

Inclusion criteria: (a) all patients had pathologically or radiologically (contrast-enhanced CT) confirmed unresectable HCC (*BCLC* stage B or C) based on the European Association for the Study of the Liver (EASL) diagnostic criteria [3]. (b) Patients with unresectable HCC treated with LRT (repeated TACE or combination of TACE and RFA) combined with sorafenib who had achieved a CR (A CR in HCC patients positive for AFP was assessed based on radiologic criteria according to mRECIST [16] and AFP normalization [17, 23]; CR in HCC patients negative for AFP was assessed based on radiologic criteria according to mRECIST. The patients who did not achieve a CR, those with a secondary malignancy, those with previous liver transplantation, and those with missing data were excluded. When a CR was observed, the discontinuation of sorafenib was recommended by the attending physician. If the patient agreed to the physician's recommendation, sorafenib was discontinued after the CR. Sorafenib was continued after the CR in patients who disagreed to the recommendation.

### Protocols for LRT

#### TACE procedure

Briefly, 10 to 20 mL lipiodol (Guerbet, Paris, France) was mixed with 20–40 mg epirubicin (Pfizer, New York, USA) to create an emulsion. Depending on the tumor size and liver function, 2–20 mL of the emulsion was infused into the liver tumor through a catheter. Subsequently, embolization using gelfoam was carried out. When blood flow slowed or a vascular cast was observed, the injection was stopped. The tumor-feeding artery was selected or super-selected whenever possible [11].

#### Radiofrequency ablation

RFA [24] was performed by using the RFA system (Cool-Tip Valleylab, USA) under real-time ultrasound guidance. After administration of analgesia (50–60 mg of propofol and 0.05−0.1 mg of fentanyl and local anaesthesia 5−15 mL of 2% lidocaine) by an anaesthesiologist, a 17-Ga RFA needle was inserted into the tumor. Each radiofrequency energy application lasted for 10 min, with the tissue temperature reaching 90°C for at least 12 min.

### Adjuvant sorafenib treatment

Generally, sorafenib treatment was started 1–3 days after LRT. The initial dose of oral sorafenib was 400 mg given twice daily [11]. Administration was suspended on the day procedure was performed and was resumed the next day. Doses were modified depending on toxicity, according to the National Cancer Institute's Common Terminology Criteria for Adverse Events, version 3.0. The sorafenib dose was reduced to 400 mg once a day for patients grade 3 toxicity. Continued administration of sorafenib was encouraged if the side-effects were manageable.

### Follow-up

Contrast-enhanced CT scan of the liver and AFP were performed 4–6 weeks after TACE or RFA to evaluate the effect of the treatment. If there was still residual viable tumor or new lesions had formed, additional TACE or RFA was performed. If patients had more than 3 tumors, or the size of a tumor was more than 3 cm, TACE was administered first. If patients with a CR to liver lesions, a further contrast-enhanced MRI of head, a chest CT scan and bone scintigraphy were performed to make sure there was no extrahepatic spread. In patients with a CR, the follow-up was performed every 2 months in the first 2 years. The follow-up interval was extended to every 3 months from 2 to 4 years after treatment and to every 6 months after 4 years. At each follow-up session, contrast-enhanced CT scan of abdomen, chest radiography, liver function test and AFP were performed. When recurrence was detected, the patients were selected for TACE, RFA, or conservative treatment, based on the number of tumors or the size or the site of tumor, the liver function tests, and the general condition of the patient.

### Assessments

In HCC patients negative for AFP (a baseline AFP level < 20 ng/mL), treatment efficacy was assessed based on radiological evaluation according to mRECIST (complete response = disappearance of any intratumoral arterial enhancement in all target lesions). In HCC patients positive for AFP (a baseline AFP level > 20 ng/mL), CR was assessed based on radiological evaluation and AFP normalization (AFP level < 20 ng/mL) [17]. Recurrence-Free Survival (RFS) was defined as the time from the first CR until radiologically confirmed recurrence or presence of a new lesion. Post-complete response overall survival (pOS) was defined as the time from the first CR until death or the last follow-up. Overall survival (OS) was defined as the time from the first local treatment until death or the last follow-up.

Quality of life was assessed by symptom scores using the 18-item hepatobiliary cancer subscale (HepCS) of Functional Assessment of Cancer Therapy-Hepatobiliary (FACT-Hep) [25] at one month after administration of sorafenib and 2 months after CR, respectively. HepCS assesses specific symptoms of hepatobiliary cancer and side-effects of treatment, which contains 18 items with a subscale score of 0–72 points. The patients scored themselves and collected by a trained research assistant. The QoL scores at the two time points were compared between the two groups, respectively.

### Statistical analysis

All statistical analyses were performed using SPSS software (version 16.0, SPSS, Chicago, IL). For baseline characteristics, continuous variables are described as medians ± standard deviation and categorical variables are expressed as frequencies and percentages. The *t* test was used to compare continuous variables between the two groups. The *χ^2^* test was used to compare categorical variables between the two groups. The Kaplan-Meier method was used to calculate the RFS, pOS and OS. Log-rank test was used to determine the difference between the two groups. All statistical tests were two-sided, and *P* < 0.05 was considered statistically significant.
